# Antimicrobial activity of β-lactam antibiotics against pneumococcal isolates causing pneumococcal disease in adults immediately before and after the COVID-19 pandemic in Spain (2019–2020)

**DOI:** 10.3389/fphar.2025.1658431

**Published:** 2025-09-30

**Authors:** Mirella Llamosí, Covadonga Pérez-García, Aída Úbeda, Erick J. Vidal-Alcántara, Inés Pareja-Cerbán, Elena Gómez-Rubio, Pilar Coronel, Juan Carlos Sanz, Mirian Domenech, Julio Sempere, Jose Yuste

**Affiliations:** ^1^ Spanish Pneumococcal Reference Laboratory, National Center for Microbiology, Instituto de Salud Carlos III, Madrid, Spain; ^2^ CIBER de Enfermedades Respiratorias (CIBERES), Instituto de Salud Carlos III, Madrid, Spain; ^3^ Scientific Department, Meiji Pharma Spain, Alcala de Henares, Spain; ^4^ Regional Public Health Laboratory, Comunidad de Madrid, Madrid, Spain; ^5^ CIBER de Epidemiología y Salud Pública (CIBERESP), Instituto de Salud Carlos III, Madrid, Spain

**Keywords:** AMR, PCV21, PCV20, serotype 11A, serotype 19A, serotype 24F, serotype 14, serotype 9V

## Abstract

**Background:**

*Streptococcus pneumoniae* remains a major respiratory pathogen. Although pneumococcal conjugate vaccines (PCVs) have reduced the burden of resistant vaccine-covered serotypes, serotype replacement has led to the emergence of non-vaccine types.

**Methods:**

This study assessed the activity of β-lactam antibiotics against 1,018 clinical isolates of *S. pneumoniae* with reduced penicillin susceptibility collected across Spain in 2019–2020. Minimum inhibitory concentrations (MICs) were determined for oral cephalosporins (cefditoren, cefuroxime, cefixime, cefpodoxime), intravenous cephalosporins (cefotaxime, cefuroxime), and penicillins (penicillin, amoxicillin). MIC_50_ and MIC_90_ values were analyzed by year, serotype (PCV20- vs. PCV21-specific), and clinical presentation.

**Results:**

Cefditoren showed the lowest MIC_50_/_90_ values among oral β-lactams, and cefotaxime was the most active intravenous cephalosporin. Most isolates were resistant to cefuroxime (oral and intravenous) based on EUCAST breakpoints. While penicillin derivatives showed susceptibility with increased exposure at MIC_50_, MIC_90_ values indicated resistance to oral amoxicillin. A rise in antibiotic use during the COVID-19 pandemic’s first year was associated with an increased proportion of resistant isolates, particularly among non-invasive respiratory infections. Serotype analysis revealed that PCV20-specific types (9V, 14 and 19A) and shared PCV20 and PCV21 serotypes (11A and 19A) had higher MIC_90_ values and bigger proportion of resistant strains to β-lactams than PCV21-unique ones (15A, 23B and 24F).

**Conclusion:**

Despite the overall increase in resistance, cefditoren and cefotaxime retained favorable *in vitro* activity, while cefuroxime, cefixime, and cefpodoxime exhibited poor efficacy. Newer PCVs may broaden protection against emerging resistant serotypes, yet continued antimicrobial resistance surveillance is essential, especially regarding non-vaccine serotypes. These findings support cefditoren and cefotaxime as viable therapeutic options against non-susceptible *S. pneumoniae* isolates.

## 1 Introduction

The use of pneumococcal conjugate vaccines (PCVs) in children and adults has proven to be an effective intervention for controlling the burden of both invasive and non-invasive pneumococcal disease (Bertran et al., 2024; Pérez-García et al., 2024; Sempere et al., 2024; Pérez-García et al., 2025), in addition to being a key measure in reducing the impact of antimicrobial resistance (AMR). In Spain, the effectiveness of PCVs (PCV7 and PCV13) in controlling the spread of resistant pneumococcal isolates has been demonstrated, and it is suggested that incorporating these vaccines into national immunization schedules is a cost-effective strategy to counteract antibiotic resistance (Sempere et al., 2022; Calvo-Silveria et al., 2025). Before the introduction of PCVs in Spain, most cases of invasive pneumococcal disease (IPD) associated with antimicrobial resistance were caused by serotypes included in conjugate vaccines (Fenoll et al., 2015). Following the implementation of PCV7 for a brief period and later PCV13, there was a drastic decline in IPD cases caused by vaccine serotypes associated with resistance (Sempere et al., 2022). However, the emergence of non-vaccine serotypes harboring antibiotic resistance has also been observed. These serotypes have been increasing in recent years, and some are associated with hypervirulence, such as the ST6521 clone in serotype 11A (Aguinagalde et al., 2015; González-Díaz et al., 2020) and the GPSC10 genetic lineage in serotype 24F (Lo et al., 2022), which is linked to severe meningitis episodes.

In the United States, PCVs have also shown an impact in reducing the burden of IPD caused by antibiotic-resistant serotypes (Bajema et al., 2022). Among children under 5 years old, the incidence of resistance-associated IPD decreased across all antibiotic classes from 2009 to 2018, dropping from 8.8 to 2.3 per 100,000 inhabitants for macrolides, from 5.9 to 0.4 per 100,000 for cephalosporins, and from 5.2 to 0.4 per 100,000 for penicillins (Bajema et al., 2022). Among adults over 65 years old during the same period, the incidence decreased from 10.4 to 7.3 per 100,000 for macrolides, from 6.2 to 2.4 per 100,000 for cephalosporins, and from 3.6 to 1.6 per 100,000 for penicillins (Bajema et al., 2022). Nearly two decades after the introduction of PCVs in the U.S., their impact on reducing IPD by AMR resistant isolates remains significant.

However, recently approved and implemented vaccines, such as PCV15 and PCV20, along with PCV21—an adult-targeted vaccine already approved by the FDA and EMA—are expected to expand coverage against non-vaccine serotypes associated with antibiotic resistance. To ensure their effectiveness, these vaccines must be administered with high coverage rates. Another problem with AMR and IPD is the possibility of increased resistant rates during the first pandemic year due to the extensive use of azithromycin and other antibiotics to prevent complications related to bacterial coinfections in patients with COVID-19 (Meschiari et al., 2022). Several authors have warned that antibiotic resistance has expanded because of the careless use of antibiotics in the medical field, the food industry, agriculture, and other industries (Singh, 2024). In addition, other studies have stated that antimicrobial resistance has emerged as a hidden pandemic following COVID-19 (Singh and Sodhi, 2023). A recent report from United States confirmed that multidrug resistant infections in hospitals increased during the pandemic due to antibiotic exposure (Yek et al., 2025).

In this study, we show the evolution of antimicrobial resistance to different β-lactam antibiotics including oral cephalosporins, intravenous cephalosporins, and penicillins, in pneumococcal isolates causing disease in adults. We compared data from 2019 to 2020, coinciding with the years before and after the COVID-19 pandemic, to assess the impact of the pandemic in the antibiotic susceptibility of *Streptococcus pneumoniae*.

## 2 Materials and methods

### 2.1 Study design

All pneumococcal isolates tested were received at the Spanish Pneumococcal Reference Laboratory (SPRL) as part of the daily routine surveillance conducted in the laboratory. The study period covers the years 2019, a late PCV13 vaccine period prior to COVID-19 pandemic and 2020, the first year of COVID-19 pandemic when non-pharmacological measures were implemented since February.

We randomly selected a minimum of 500 clinical isolates from 2019 to 2020 that were non-susceptible to penicillin with a minimum inhibitory concentration (MIC) ≥ 0.12 mg/L. In this case, we selected 500 of 586 non-susceptible isolates from 2019 (85.3%), and all 518 non-susceptible isolates from the year 2020 (100%). The 2019 isolates were randomly chosen from our nationwide collection, which already ensures representative sampling across Spanish regions. As our study focused on characterizing the overall population of penicillin non-susceptible isolates rather than regional differences, random selection was considered sufficient to maintain representativeness, while ensuring that the final selection still included isolates from all regions. All clinical isolates were obtained from adult patients, with an average age of 57 years in 2019, and 61 years in 2020. Isolates were categorized into three clinical groups: invasive pneumonia (blood isolates from hospitalized pneumonia patients), non-invasive pneumonia (bronchoaspirate isolates from pneumonia cases), and non-respiratory invasive infections (blood isolates from non-pneumonia cases). Meningitis isolates were excluded from the study. Pneumonia was diagnosed by clinicians based on clinical criteria and recorded using the International Classification of Diseases (ICD) codes. Cases were reported to the pneumococcal reference laboratory when strains were submitted for characterization from the different Spanish hospitals. All respiratory invasive pneumonia strains were isolated from hemocultures, whereas non-invasive pneumonia strains were obtained from bronchoaspirates. For non-respiratory invasive infections, all strains were isolated from patients without respiratory disease and were also obtained from hemocultures.

The SPRL annually reports all IPD cases in Spain to the European Center for Disease Control (ECDC), covering up to 80%–85% of the national cases according to estimates from the National Center for Epidemiology. Since 2018, it has also reported cases to the Invasive Respiratory Infection Surveillance Network (IRIS). Pneumococcal identification was confirmed using the Optochin-susceptibility test and bile solubility test. Serotyping was conducted using the Quellung reaction, dot blot assay with specific antisera, and/or PCR-capsular sequence analysis as previously described (de Miguel et al., 2021; Pérez-García et al., 2024). Optochin disk and blood agar plates were purchased from Becton Dickison Spain. Antipneumococcal sera for serotyping were purchased from Statem Serum Institute (Denmark).

### 2.2 Antimicrobial susceptibility testing

We included and compared antimicrobial susceptibility testing of oral cephalosporins (cefixime, CFX; cefditoren, CDN; cefpodoxime, CPD; and cefuroxime, CXM), intravenous cephalosporins (cefotaxime, CTX; cefuroxime, CXM) and other β-lactams (penicillin, PEN; amoxicillin, AMX). MICs were determined by agar dilution technique following EUCAST clinical breakpoints from 2023 (Table 1).

**TABLE 1:** MIC breakpoints for *Streptococcus pneumoniae* considered in the study following EUCAST criteria.

	MIC breakpoints (mg/L)	
	S ≤	R >
Cefixime	0.5	2
Cefpodoxime	0.25	0.5
Cefditoren	0.5	2
Cefuroxime oral	0.25	0.25
Cefotaxime	0.5	2
Cefuroxime i.v	0.5	1
Penicillin	0.06	2
Amoxicillin	0.5	1

*Streptococcus pneumoniae* ATCC 49619 standard strain was used as control. Antimicrobials were purchased from Sigma Aldrich (Merck, United States). Cefditoren was obtained from Meiji Pharma Spain.

### 2.3 Statistical analysis

Data of antimicrobial susceptibility to each antibiotic are representative of results obtained from all the isolates analyzed of each year (500 isolates from 2019 and 518 from 2020). Statistical analysis was performed by using two-tailed Student’s t-test (for two groups’ comparisons). GraphPad InStat version 8.2 (GraphPad Software, San Diego, CA, United States) was used for data analysis. Differences were considered statistically significant with P < 0.05 (*) and highly significant with P < 0.01 (**) and P < 0.001 (***). MIC_50_ represents the minimum concentration of an antimicrobial that inhibits 50% of the tested isolates, while MIC_90_ represents the concentration that inhibits 90% of the isolates, and they were calculated as the 50th and 90th percentiles respectively.

## 3 Results

### 3.1 Cefditoren and cefotaxime are the cephalosporins with lower MIC values during the study period

**FIGURE 1:**
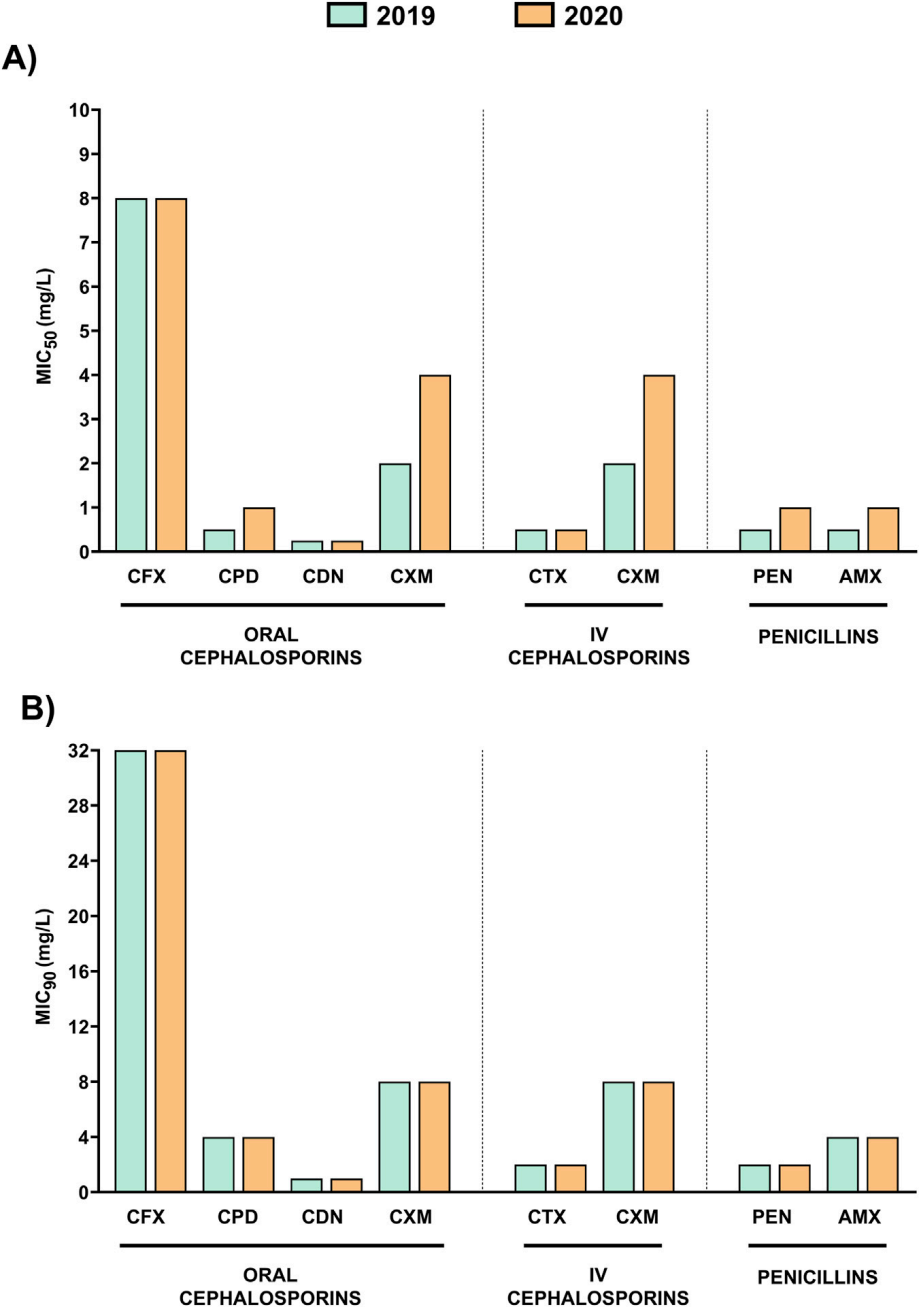
Comparison of MIC_50_ and MIC_90_ values for oral cephalosporins, intravenous cephalosporins, and penicillins in 2019 and 2020. **(A)** MIC_50_ values (mg/L) for all isolates tested in the study in 2019 (green) and 2020 (orange). **(B)** MIC_90_ values (mg/L) for all isolates tested in the study in 2019 (green) and 2020 (orange).

Analysis of antimicrobial susceptibility of oral cephalosporins confirmed that cefixime exhibited the highest MIC_90_ and MIC_50_ values in 2019 and 2020, with values of 32 mg/L and 8 mg/L, respectively, in both years (Figure 1). These non-susceptibility levels corresponded to resistance according to EUCAST breakpoints. Cefuroxime was the second oral cephalosporin, with the highest MIC_90_ and MIC_50_ values in 2019–2020. Its MIC_50_ value increased from 2 mg/L in 2019 to 4 mg/L in 2020, while its MIC_90_ remained at 8 mg/L in both years (Figure 1). All MIC values for cefuroxime classified the studied isolates as resistant according to both oral and intravenous EUCAST breakpoints (Table 1). Cefpodoxime presented MIC_90_ and MIC_50_ values of 4 mg/L and 0.5–1 mg/L, respectively being classified as resistant with EUCAST breakpoints. Cefditoren had MIC_90_ and MIC_50_ values of 1 mg/L and 0.25 mg/L, respectively, in both years (Figure 1). This is interesting because cefditoren showed the lowest MIC values among all oral cephalosporins studied.

Among intravenous cephalosporins, cefotaxime presented lowest MIC values than cefuroxime, with MIC_90_ of 2 mg/L and MIC_50_ of 0.5 mg/L in 2019–2020 (Figure 1). Antibiotic interpretation according to EUCAST criteria, showed that pneumococcal isolates may be considered susceptible with increased exposure to cefotaxime and resistant to cefuroxime (Table 1). Statistical analysis confirmed significant differences for cefditoren as the antibiotic with the lowest MIC in comparison to the rest of β-lactam antibiotics including cefotaxime (***P < 0.001, two-tailed Student’s t-test).

**FIGURE 2:**
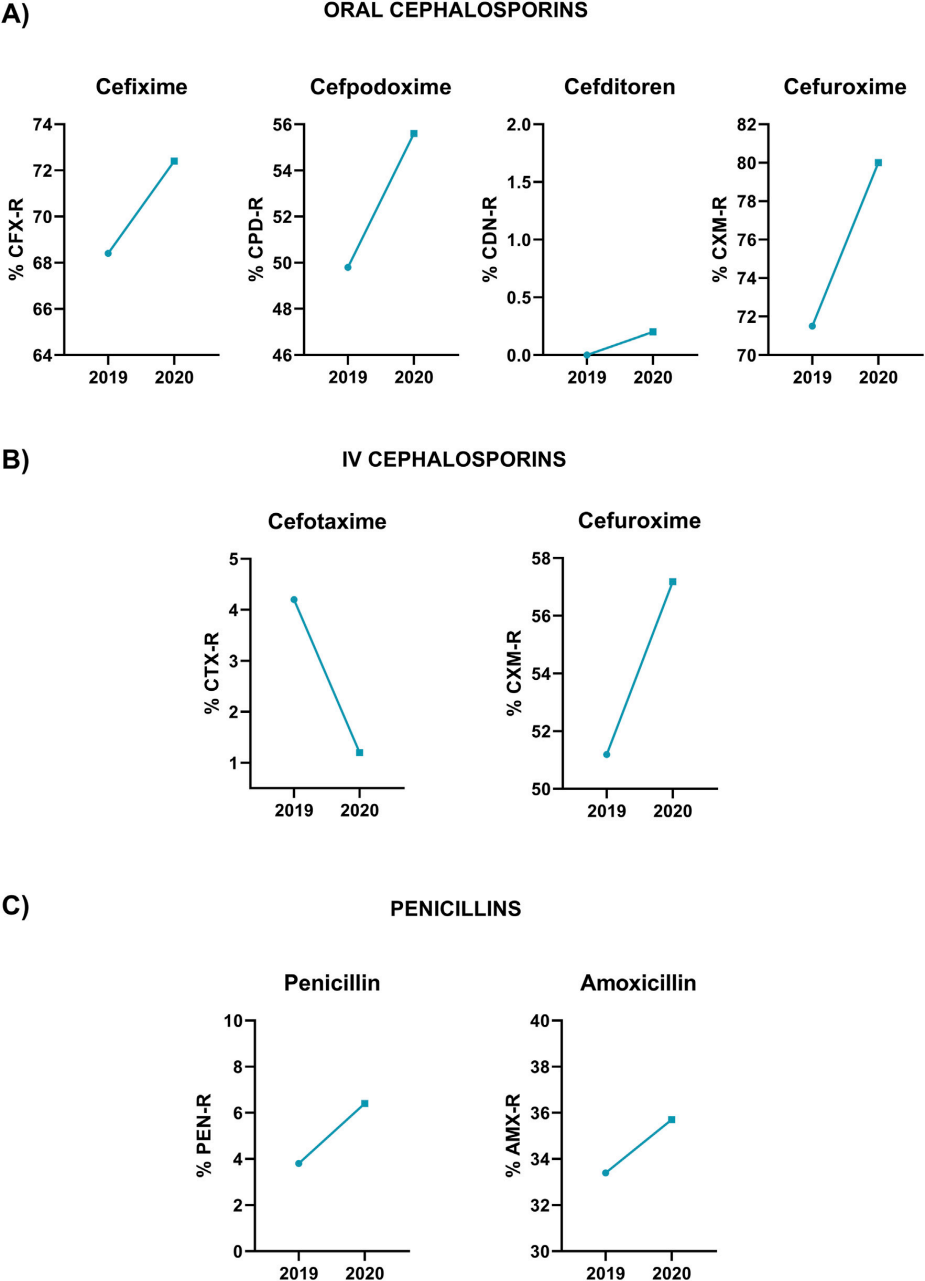
Proportion of resistant isolates in 2019 and 2020 following EUCAST clinical breakpoints to the different antibiotics tested in the study. **(A)** Oral cephalosporins. **(B)** Intravenous cephalosporins. **(C)** Penicillins.

In most cases, general MIC values were similar between 2019 and 2020 but for cefuroxime, cefpodoxime, penicillin and amoxicillin, we observed an increase in the MIC_50_ value from 2019 to 2020 (Figure 1A). In the case of amoxicillin, this represents a shift from “susceptible with increased exposure” to “resistant” (Table 1). Moreover, to evaluate the impact of higher antibiotic consumption at the early stages of COVID-19 pandemic, we evaluated the proportion of pneumococcal resistant isolates in 2019 and 2020 (Figure 2). Our results showed an increased pattern of antibiotic resistance to most antibiotics evaluated from 2019 to 2020 (Figure 2). The increase was remarkable for all antibiotics but for cefditoren and cefotaxime, where we only observed a slight increase in the proportion of resistant isolates of 0.2% or a diminish in the proportion of resistance isolates to 1.2% respectively (Figure 2).

### 3.2 Cefditoren and cefotaxime maintain low MIC values regardless of the source of the infection

The outcome of the infection based on the antibiotic treatment choice may be affected by the localization of the infectious process. Infections of the respiratory tract that may be caused in many cases by bacteria forming biofilms such as patients with chronic obstructive pulmonary disease (COPD) might be more difficult to eradicate than bacteria from other origins that are not associated with biofilms (Kyd et al., 2011; Domenech et al., 2012). Invasive isolates (regardless of the origin) had lower MIC values for intravenous cephalosporins (cefotaxime and cefuroxime) and penicillins (penicillin and amoxicillin) in comparison to non-invasive isolates from respiratory origin (Table 2). Another interesting finding was that cefditoren and cefotaxime as oral and intravenous options respectively showed the lowest MIC_90_ values (0.5–1 mg/L) of all antibiotics tested (Table 2).

**TABLE 2:** *In vitro* activity of oral cephalosporins, intravenous cephalosporins, and penicillins in clinical isolates from different sources. Number of isolates from each clinical group are indicated in the table.

Antibiotics		MIC_90_ (mg/L)	
		2019	2020
		Invasive pneumonia	
		n= 246	n= 286
Oral cephalosporins	Cefixime	32	32
	Cefpodoxime	4	4
	Cefditoren	1	1
	Cefuroxime	8	8
Intravenous cephalosporins	Cefotaxime	2	2
	Cefuroxime	8	8
penicillins	Penicillin	2	2
	Amoxicillin	4	4

Antibiotics		Invasive non-respiratory	
		n= 197	n= 146
Oral cephalosporins	Cefixime	32	16
	Cefpodoxime	4	2
	Cefditoren	1	0.5
	Cefuroxime	8	8
Intravenous cephalosporins	Cefotaxime	2	1
	Cefuroxime	8	8
Penicillins	Penicillin	2	2
	Amoxicillin	4	4

Antibiotics		Non-invasive pneumonia	
		n= 57	n= 86
Oral cephalosporins	Cefixime	32	32
	Cefpodoxime	4	4
	Cefditoren	1	1
	Cefuroxime	8	8
Intravenous cephalosporins	Cefotaxime	4	2
	Cefuroxime	8	8
Penicillins	Penicillin	4	4
	Amoxicillin	8	8

### 3.3 Evolution of antibiotic resistance in prevalent unique serotypes of PCV20 and PCV21 causing IPD in adults

**FIGURE 3:**
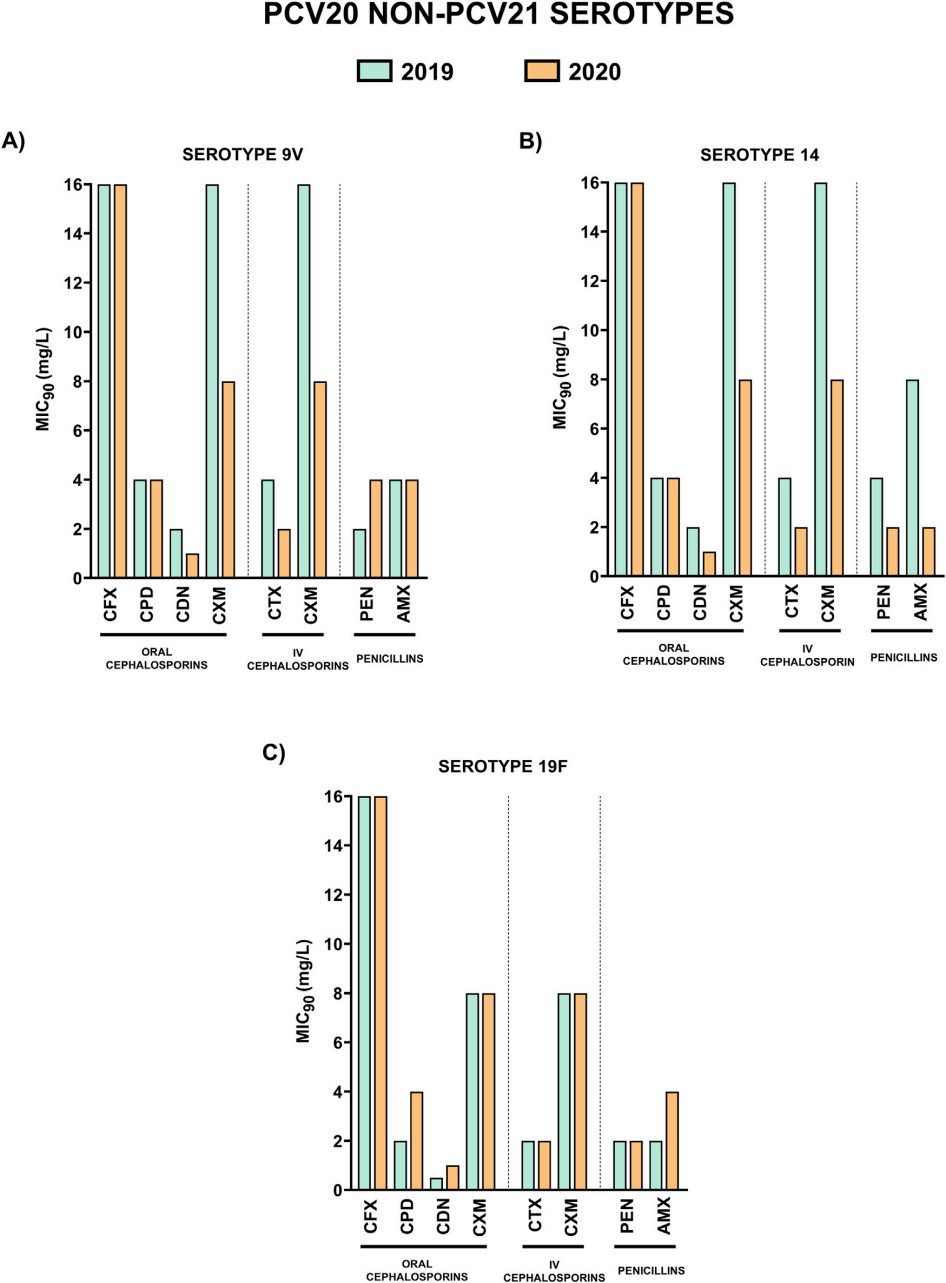
Comparison of MIC_90_ values of oral cephalosporins, intravenous cephalosporins and penicillins for the most prevalent serotypes associated with non-susceptibility to penicillin included in PCV20 and not in PCV21. 2019 is shown in green and 2020 in orange. **(A)** MIC_90_ values (mg/L) for serotype 9V. **(B)** MIC_90_ values (mg/L) for serotype 14. **(C)** MIC_90_ values (mg/L) for serotype 19F.

**FIGURE 4:**
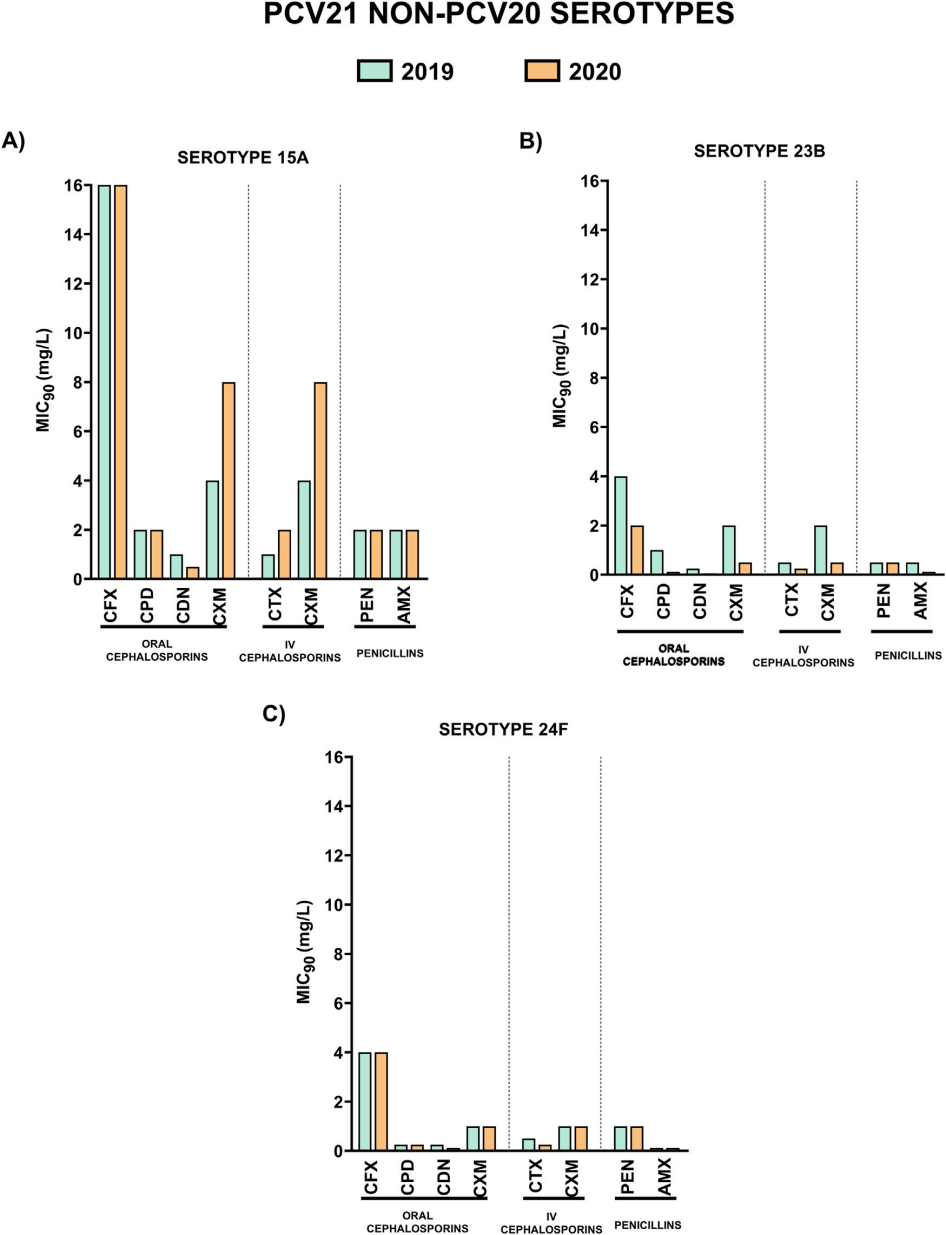
Comparison of MIC_90_ values of oral cephalosporins, intravenous cephalosporins and penicillins for the most prevalent serotypes associated with non-susceptibility to penicillin included in PCV21 and not in PCV20. 2019 is shown in green and 2020 in orange. **(A)** MIC_90_ values (mg/L) for serotype 15A. **(B)** MIC_90_ values (mg/L) for serotype 23B. **(C)** MIC_90_ values (mg/L) for serotype 24F.

**FIGURE 5:**
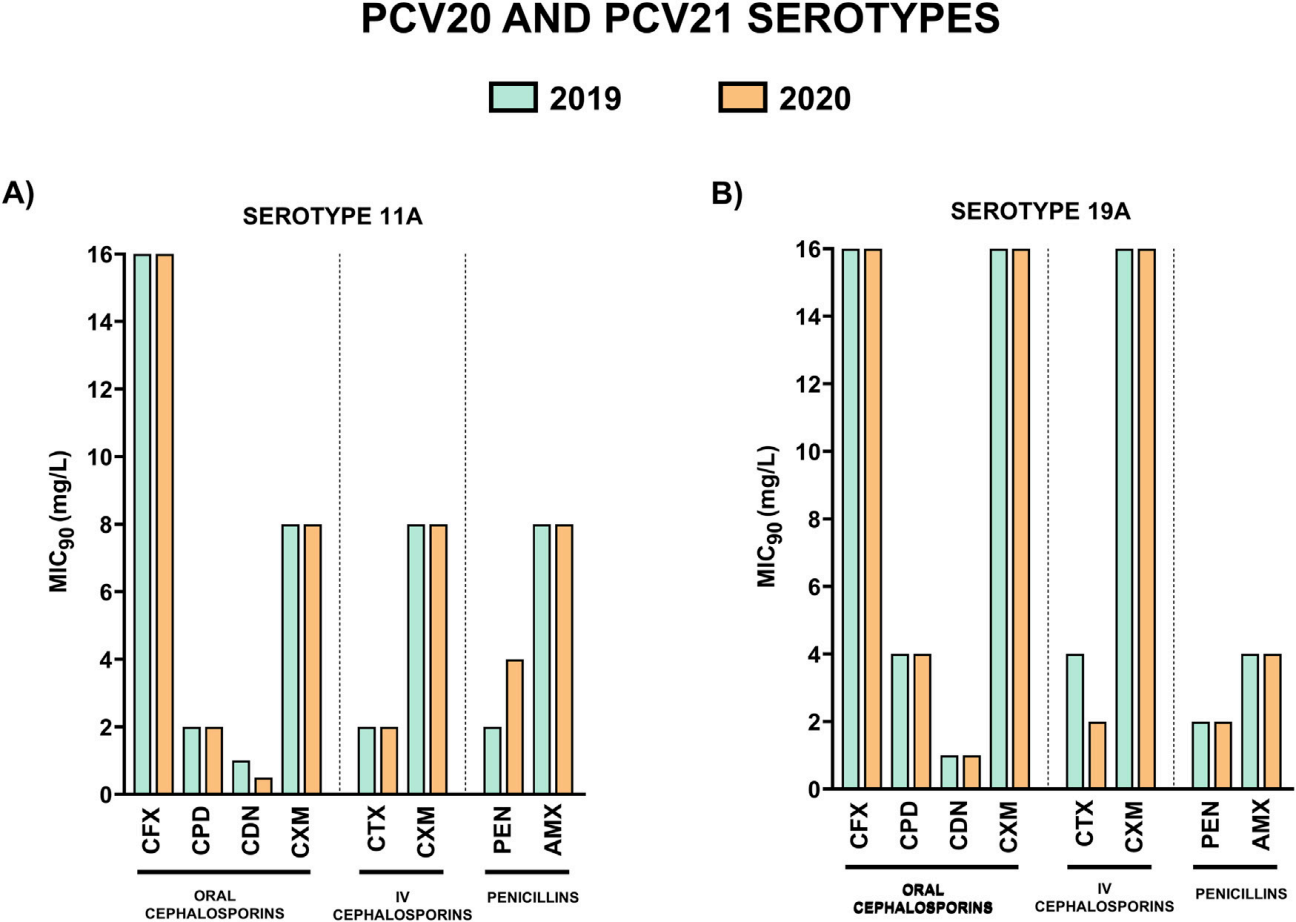
Comparison of MIC_90_ values of oral cephalosporins, intravenous cephalosporins and penicillins for the most prevalent serotypes associated with non-susceptibility to penicillin included in both PCV20 and PCV21. 2019 is shown in green and 2020 in orange. **(A)** MIC_90_ values (mg/L) for serotype 11A. **(B)** MIC_90_ values (mg/L) for serotype 19A.

Characterization of the resistance levels of serotypes included in the recent approved vaccines PCV20 and PCV21 is essential to evaluate their impact to prevent pneumococcal disease caused by resistant isolates of these serotypes. Hence, we analyzed the MIC_90_ values for the different β-lactams of the most prevalent unique PCV20 serotypes that are not included in PCV21 (9V, 14 and 19F), unique PCV21 serotypes not included in PCV20 (15A, 23B and 24F) and common PCV20/PCV21 serotypes that are present in both vaccines (11A and 19A) (Figures 3–5).

Unique PCV20 serotypes and those that are shared by both vaccines, presented higher MIC_90_ values to the different antibiotics in comparison to unique PCV21 serotypes (Figures 3, 4). Among oral cephalosporins, cefixime exhibited the highest MIC_90_ values in all the serotypes studied followed by cefuroxime and cefpodoxime. In contrast, cefditoren demonstrated the greatest activity across all serotypes, with the lowest MIC_90_ values, even considering the intravenous cephalosporin cefotaxime (Figures 3–5). When studying the activity against penicillins, unique PCV20 serotypes and common PCV20/PCV21 serotypes presented higher MIC_90_ levels to both penicillin and amoxicillin (Figures 3, 5). Our study confirmed that serotype 11A that is present in PCV20 and PCV21 had the higher MIC_90_ levels to penicillin and amoxicillin (Figure 5). Moreover, we also observed that unique PCV20 serotypes, especially serotypes 9V and 14, and common PCV20/PCV21 serotypes (11A and 19A) presented a higher proportion of resistant strains to the different β-lactams than unique PCV21 serotypes (Table 3). None of the unique PCV21 non-PCV20 serotypes presented resistant strains to penicillin or cefotaxime, and only serotype 15A presented strains resistant to amoxicillin (Table 3). Cefditoren was the only β-lactam without resistant strains in the most prevalent serotypes associated with non-susceptibility to penicillin in Spain.

**TABLE 3:** Percentage of resistant isolates to β-lactams for the different serotypes analyzed in the study. Serotypes associated with non-susceptibility to penicillin included in PCV20 and not in PCV21 (9V, 14 and 19F). Serotypes associated with non-susceptibility to penicillin included in PCV21 and not in PCV20 (15A, 23B and 24F). Serotypes associated with non-susceptibility to penicillin included in both PCV20 and PCV21 (11A and 19A).

Antibiotics		PCV20 non-PCV21 serotypes						PCV21 non-PCV20 serotypes						PCV20 and PCV21 serotypes			
		Serotype 9V		Serotype 14		Serotype 19F		Serotype 15A		Serotype 23B		Serotype 24F		Serotype 11A		Serotype 19A	
		2019	2020	2019	2020	2019	2020	2019	2020	2019	2020	2019	2020	2019	2020	2019	2020
Oral cephalosporins	Cefixime	100	100	100	92.3	72	95	78.8	66.7	18	0	92.9	90.6	97.8	95.1	79.2	89.6
	Cefpodoxime	90.9	93.8	97.2	89.7	68	95	60.6	40	10	0	1.2	0	94.4	95.1	77.9	85.4
	Cefditoren	0	0	0	0	0	0	0	0	0	0	0	0	0	0	0	0
	Cefuroxime	100	100	100	97.4	72	95	84.8	80	30	15.4	96.4	98.1	96.6	100	80.5	91.6
Intravenous cephalosporins	Cefotaxime	9.1	0	13.9	2.6	0	0	0	0	0	0	0	0	3.4	0	9.1	4.2
	Cefuroxime	100	100	97.2	97.4	60	90	57.6	40	16	0	3.6	1.9	95.5	96.7	75.3	87.5
Penicillins	Penicillin	0	6.3	13.9	5.1	0	5	0	0	0	0	0	0	4.5	26.2	6.5	8.3
	Amoxicillin	81.8	81.3	36.1	38.5	20	35	15.2	6.7	0	0	0	0	92.1	91.8	70.1	64.5

**FIGURE 6:**
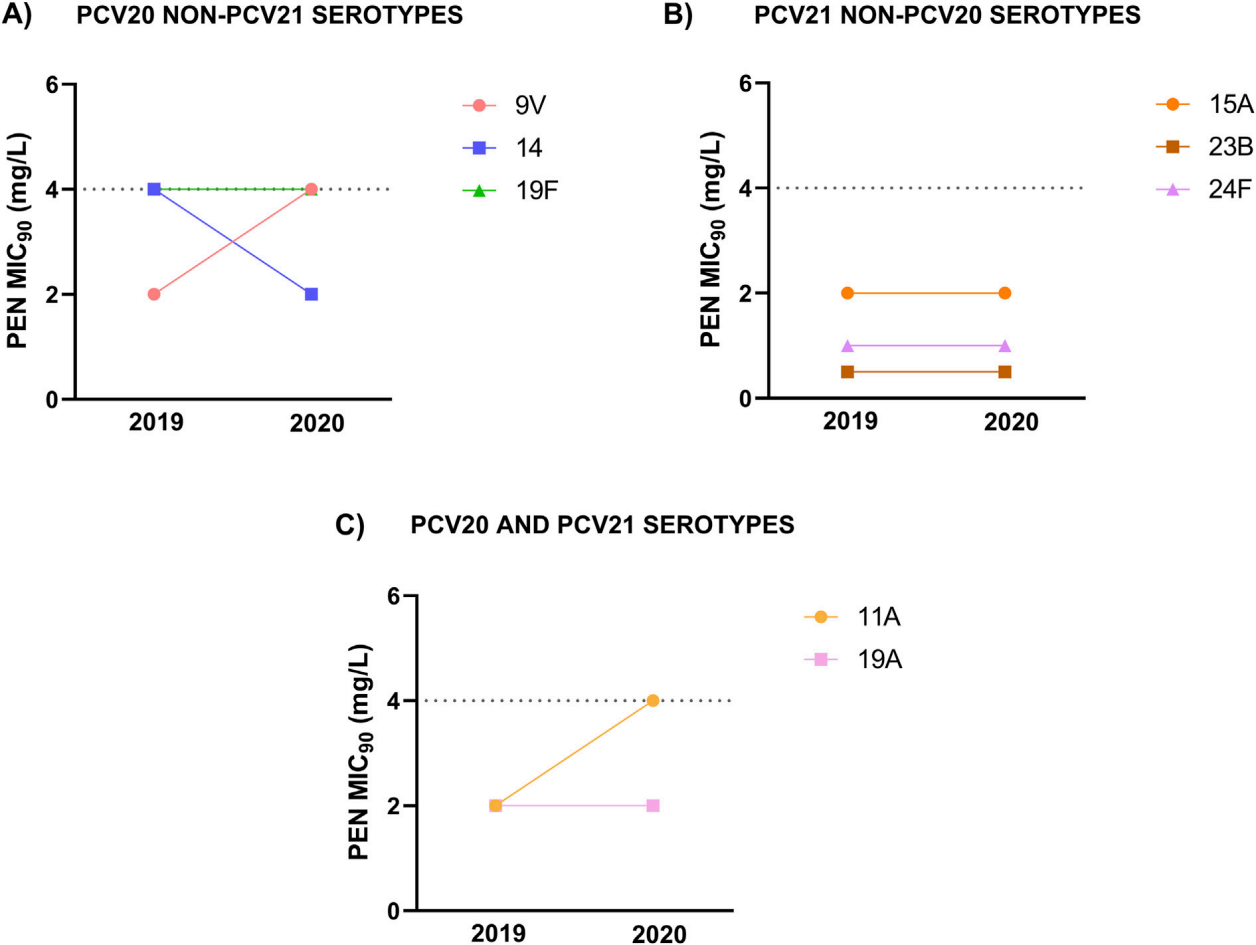
Penicillin MIC_90_ values in 2019 and 2020 for the different serotypes analyzed in the study. **(A)** Serotypes associated with non-susceptibility to penicillin included in PCV20 and not in PCV21. **(B)** Serotypes associated with non-susceptibility to penicillin included in PCV21 and not in PCV20. **(C)** Serotypes associated with non-susceptibility to penicillin included in both PCV20 and PCV21.

When studying the specific impact of higher antibiotic consumption during the early stages of the COVID-19 pandemic on resistance patterns in serotypes, we observed changes in some of them between 2019 and 2020. This was of special relevance for certain serotypes such as 9V (unique to PCV20) and 11A (PCV20/PCV21) changing the consideration to penicillin by EUCAST from ‘susceptible with increased exposure’ in 2019 (MIC_90_; 2 mg/mL) to ‘resistant’ in 2020 (MIC_90_; 4 mg/mL) (Figure 6). This was also highlighted in the rise of the proportion of resistant strains to penicillin in 2020 for serotypes 9V and 11A, from 0% to 6.3% and from 4.5% to 26.2% respectively (Table 3).

## 4 Discussion

A key breakthrough in combating pneumococcal disease was the development of antibiotic chemotherapy, which emerged in the 1930s and led to the discontinuation of serum therapy. Nevertheless, the effectiveness of antibiotic chemotherapy was soon threatened by the emergence of resistant clinical isolates, which appeared shortly after the widespread use of antibiotics (Spagnolo et al., 2021). The prevalence of antibiotic resistance in pneumococcus increased globally in the pre-PCV era, and despite the current use of vaccines with high coverage rates in pediatric population, the rise of serotypes associated with antibiotic resistance remains as a concerning phenomenon (Cassiolato et al., 2018; Sempere et al., 2022). Resistance patterns vary depending on serotype and geographic region, as several factors contribute to the emergence and spread of resistant isolates, including low vaccination rates specially in adult population, serotype replacement by non-vaccine serotypes, and inappropriate antibiotic use (Reinert, 2009; Song et al., 2012; Chávez et al., 2021). However, prophylactic strategies based on immunization appear to be the best approach for controlling the development of IPD and pneumonia, as well as reducing the impact of antibiotic resistance (Atkins et al., 2018).

Our results showed a rise in antibiotic resistance during the first year of the COVID-19 pandemic. During 2020, the generic use of antibiotics to avoid co-infections with bacterial pathogens was extended (Langford et al., 2021) and antibiotic sales correlated positively with COVID-19 cases globally (Nandi et al., 2023). Moreover, a recent United States study confirmed that AMR infections in hospitalized patients during the pandemic increased due to antibiotic exposure (Yek et al., 2025), which is in line with our observations. This makes antimicrobial stewardship essential, even in the context of a global health crisis (Langford et al., 2021), as inadequate use of antibiotics may exacerbate the antimicrobial resistance problem (Group, 2021).

In this study we showed that cefditoren was the β-lactam antibiotic with the highest proportion of susceptible isolates, achieving the lowest MIC_50_ and MIC_90_ values among all antibiotics tested, including other oral and intravenous cephalosporins. Since its introduction in 2004 and despite its widespread use in clinical practice, the emergence of non-susceptible isolates to this antibiotic has remained very low. In contrast, cefixime, cefpodoxime and cefuroxime, two third-generation and one second-generation oral cephalosporins, had the lowest proportion of susceptible isolates throughout the study period. It had already been demonstrated that cefditoren maintains good activity against penicillin-non-susceptible isolates due to its high affinity for PBP2x, enhancing its antimicrobial activity, which explains the consistently low and stable MIC_50_ and MIC_90_ values (0.5 and 1 μg/mL in most of the clinical isolates analyzed) (Yamada et al., 2007; Yang et al., 2012).

Lower respiratory tract infections (LRTIs) are associated with high morbidity and mortality rates worldwide. Before the COVID-19 pandemic, nearly 2.4 million deaths were caused by infections affecting the LRT being *S. pneumoniae* the major etiologic agent of these cases, affecting largely to high-risk groups such as the pediatric and elderly population (children under 5 years old and adults over 65 years old) (GBD, 2018). In this sense, our findings demonstrated that cefditoren as oral option and cefotaxime as iv choice, exhibited the highest antimicrobial activities regardless of the serotype or source of the clinical isolate. This is important from the LRTIs perspective as both cephalosporins are indicated for the treatment of community-acquired pneumonia (Granizo et al., 2006; Di Marco et al., 2014). We observed higher MIC values for iv cephalosporins and penicillins in non-invasive isolates than in invasive isolates (both with or without respiratory origin). Other studies have also described a higher proportion of resistance in non-invasive isolates compared with IPD isolates (Mohanty et al., 2023). Our results demonstrated that cefditoren maintained the same antimicrobial activity regardless of the source of the clinical isolate. Cefditoren can be used as oral treatment following parenteral administration of third-generation cephalosporins such as cefotaxime or ceftriaxone (Monmaturapoj et al., 2012). This is particularly relevant because early switching from intravenous to oral antibiotic therapy in patients with severe community-acquired pneumonia has been shown to be safe and to reduce hospitalization time (Mandell et al., 2007). It is also recommended by various organizations, including the Infectious Diseases Society of America (IDSA) and the American Thoracic Society (ATS). In this context, cefditoren has been recommended as the most suitable option for the early switch from intravenous third-generation cephalosporins to oral therapy, as it has a similar spectrum of activity but greater intrinsic efficacy (Blasi et al., 2016). This transition benefits not only patients but also healthcare systems by reducing hospitalization time and costs, as well as the risk of hospital-acquired infections—one of the major healthcare challenges today. This concept agrees with a recent study evaluating the hospital burden of pneumococcal disease in Spain showing that the annual cost of pneumococcal hospitalizations for the national health system exceeded EUR 383 million (Gil-Prieto et al., 2025).

Although the introduction of conjugate vaccines significantly reduced IPD cases caused by vaccine serotypes and helped control the spread of multidrug-resistant isolates, many countries have reported serotype replacement, including the emergence of multidrug-resistant serotypes (Cassiolato et al., 2018; Ladhani et al., 2018; de Miguel et al., 2021; Ouldali et al., 2021). Especially, serotypes associated with hypervirulent lineages, such as 11A and 24F, are of great concern (Aguinagalde et al., 2015; Lo et al., 2022). Recently, new conjugate vaccines PCV15 and PCV20 have been approved and introduced in Spain and PCV21 has been recently approved by FDA and EMA. This will be very useful from the public health perspective because the use of broader vaccines would increase the potential coverage against resistant isolates. For instance, PCV20 and PCV21 include serotype 11A, that showed high MIC_90_ values for most antibiotics tested, although cefditoren and cefotaxime still maintained a good antimicrobial activity profile against this serotype. Moreover, PCV21, a vaccine targeting specifically the adult population, has been just approved by FDA and EMA, and includes non-PCV20 serotypes such as 15A, 23B and 24F, and could be a potential candidate to ameliorate the emergence of these multidrug-resistant serotypes (Sempere et al., 2022). However, PCV21 excluded PCV20 serotypes such as 14, 19F and 9V that diminished after PCV13 introduction but are still an important cause of IPD in Spain (Pérez-García et al., 2024).

To summarize, we evaluated the evolution of antimicrobial resistance to different β-lactam antibiotics by comparing the years before and after the COVID-19 pandemic, in order to assess its impact on the antibiotic susceptibility of *S. pneumoniae*. The main limitations of the manuscript are the lack of results covering recent years although this will be address in future studies, and the lack of information on patients’ clinical characteristics.

Overall, we found that cefditoren as oral cephalosporin, and cefotaxime as iv option, are good candidates for the treatment of pneumococcal disease in Spain, even when it is associated with reduced susceptibility to β-lactams. Moreover, the high antimicrobial activity of cefditoren might reinforce its use as an oral option after pneumococcal hospitalization. From the prevention perspective, the use of higher valency conjugate vaccines in Spain might tackle the emergence of non-PCV13 serotypes associated with antibiotic resistance.

## Data Availability

The raw data supporting the conclusions of this article will be made available by the authors, without undue reservation.
